# Large-Scale Embryo Transfer Operation in Dromedary Camels: Retrospective Analysis of the Association Between Key Clinical Factors and the 2-Month Pregnancy Rate

**DOI:** 10.3390/ani15131859

**Published:** 2025-06-24

**Authors:** Taher Kamal Osman, Sayed Taha Ismail, Hossam R. El-Sherbiny

**Affiliations:** 1 Theriogenology Department, Faculty of Veterinary Medicine, Cairo University, Giza 12211, Egypt; 2 Research and Development Department, BIORASCO Co., Qassim 52232, Saudi Arabia; 3 Biotechnology Department, Salam Veterinary Group Co., Qassim 52232, Saudi Arabia

**Keywords:** uterine tone, reproductive biotechnology, embryo quality, camelids, female

## Abstract

ET is used in camel reproduction to increase the reproductive potential of elite females selected for production and show. This retrospective study analyzed the association between factors related to embryo flushing (flushing fluid turbidity and debris), embryo quality (grades 1–4), recipient uterine status (tone and endometrial microcalcifications (EM)), farm and its locations (Qassim and Hail), as well high and low temperature on the likelihood of establishment of the 2-month pregnancy rate (PR) in dromedary camels. A total of 4360 embryos were transferred to 2947 recipients in this study. Logistic regression analysis (binary) was applied to evaluate the association between the selected factors and PR in month two. The likelihood of PR was affected by embryo quality (*p* < 0.01), EMs (*p* < 0.01), and farm (*p* < 0.05) and its location (*p* < 0.01). Transferring embryos to low (*p* < 0.01) or medium (*p* < 0.05) EM recipients decreased the likelihood of PR by ~1.3 times compared to uteri without EM. Grade 3 or 4 embryo transfer decreased the likelihood of PR by ~1.9 and 2.6 times, respectively, compared to grade 1 embryos (*p*< 0.01). In Saudi Arabia, applying an ET program in dromedaries in the Hail region raised the prediction of PR over the Qassim region by 1.2 times. Temperature changes had no effect on PR; however, higher temperature only affected the PR when grade 4 embryos were transferred. In conclusion, the likelihood of PR was higher with the transfer of grade 1 or 2 embryos in an EM-free uterus in the Hail region (Saudi Arabia). PR was only affected by higher temperature in the case of transferring grade 4 embryos.

## Simple Summary

Embryo transfer (ET) success in dromedary camels faces several challenges to reach fertility standards seen in bovine ET. Uterine status of the recipients, embryo quality, and environmental conditions have been considered to affect the ET success rates in other species. The evaluated parameters are either unique to camels (hatched vs. zona pellucida-enclosed embryos), rare and unrecognized in camel fertility (endometrial microcalcifications (EMs)), or may have an impact on the ET success rate in camels (uterine tone, flushing fluid quality, and environmental conditions). Furthermore, the chosen parameters were all connected to the ET day. We found that EMs, embryo quality, and environmental conditions affected the ET success rate in dromedaries. These findings help to improve ET success rates by transferring embryos of high quality into feasible recipients under optimum environmental conditions.

## 1. Introduction

The camel breeding industry has been witnessing a marked improvement to fulfill the progressive demand by investors in racing and show camels, as well as meat and milk production. Female camels commonly produce about five calves throughout their reproductive life [1]; embryo transfer (ET) provides an alternative to increase the reproductive potential of genetically elite females to about five calves per year [2,3]. Despite efforts to increase ET success rates since the 1990s, the established 2-month pregnancy rate (PR) ranges from 21 to 42% of transferred embryos, which is still lower than bovine ET programs worldwide [4,5,6,7].

There is still limited information on key factors that can affect the outcome of ET to synchronized recipients, such as factors related to recipients’ uterine status (endometrial microcalcifications and uterine tone), flushing fluid appearance (turbidity and debris), and embryo quality [2,8]. Flushing fluid turbidity reflects embryonic environment quality and procedural precision, as it may arise from blood (trauma during flushing), mucus (cervical origin), and epithelial cells (improper catheter handling). These contaminants may reduce embryo recovery rate and the PR after ET through the induction of oxidative stress or embryonic toxicity [4,9].

Embryo quality assessment is crucial for higher PRs after ET in domestic animals. Unlike bovines, camel embryo grading differs, as only hatched blastocysts are transferred for optimum success rates; therefore, exclusive quality scoring should be tailored for camels to fit the hatched blastocysts vs. morulae, early and expanded blastocysts commonly transferred in bovines. Grades 1, 2, 3, and 4 are assigned for excellent, good, bad, and folded or damaged embryos, respectively, based on the appearance of the trophectoderm, the shape of the embryo, and its transparency [3,10]. Numerous studies conducted on alpacas [8] and camels [4,6,7] guaranteed a high pregnancy rate after embryo transfer using high-grade embryos as opposed to low-grade embryos. The transfer of dark, elongated, and spindle embryos has been reported to result in low PRs after ET in dromedaries [7].

Real-time uterine tissue visualization using ultrasonography is crucial for precise evaluation of the uterine environment prior to ET [3]. This evaluation is mandatory to exclude recipients with critical problems hindering embryo development and implantation [11,12]. Endometrial microcalcifications (EMs) are benign, tiny hyperechoic foci (concentric or amorphous; stromal or glandular) mainly visualized in the endometrium and/or endocervix during ultrasonography [13]. Immunohistochemical evaluation revealed that EMs are composed of osteopontin, osteonectin, and bone sialoprotein, which are known to be involved in calcification processes such as urolithiasis [14]. These white dots may indicate aging, chronic inflammation, or degeneration of the endometrial glands as well as uterine polyps [15].

Uterine tonicity refers to uterine rigidity in response to the effect of steroid hormones [16]. The progesterone (P_4_)/estradiol (E_2_) ratio interprets the final degree of uterine tonicity. In detail, P_4_ has the affinity to suppress the E_2_ receptors at the epithelial and myometrial cells in the uterine wall; therefore, uterine tonicity has been used as a functional marker for P_4_ levels in bovines. Optimum P_4_ concentrations inhibit the E_2_ effect on myometrial cells, resulting in a flaccid uterus and vice versa [17,18]. In bovines, the predictivity of uterine tone on pregnancy outcomes has been controversial, between a non-significant [19] or significant effect [20].

In dromedaries, many factors have been noted to affect ET success rate, such as age, season, embryo recovery timing, bull effect, and more [4,5,6,7]. The selected factors were gathered as they are related to the day of ET. They are classified into donor-related factors (embryo quality, flushing media turbidity, and debris), recipient-related factors (uterine tone and EMs), and environmental factors (location, farm, and ET day high and low temperature).

Extensive literature analysis has shown that the grading of flushing fluid turbidity and debris existence, as well as uterine tone and EMs, have typically not been investigated in dromedary camels during ET. It was hypothesized that donor factors (embryo quality, flushing media turbidity, and debris), recipient factors (uterine tone and EMs), and environmental factors (location, farm, temperature changes) would affect the PR after ET in dromedaries. Therefore, this retrospective study examined the likelihood of flushing fluid turbidity and debris, as well as uterine tone and EM, affecting the established PR in the dromedary camel ET program. Moreover, the effect of embryo quality, ET day high and low temperature, farm, and the location where the ET program has been applied were investigated.

## 2. Materials and Methods

### 2.1. Camels and Housing

Camel donors (non-lactating; n = 493) and bulls (n = 65), both aged 6 to 15 years, in addition to 2947 recipients, were used in the current retrospective study (season 2022–2023). All camels were cleared for breeding during a gynecological and andrological assessment at the beginning of the reproductive season (November-April). They were kept in sand-grounded closed barns on 5 different farms: 3 of them located in Buraydah, Qassim, Saudi Arabia (26°20′00″ N 43°58′00″ E), and the other 2 located in the Hail region, Saudi Arabia (27°31′33″ N 41°40′29″ E). Five groups of vets completed the daily work on the different farms. All locations share similar protocols for hormonal treatments, nutrition, and ET procedures. Their daily feed was composed of cubes (crushed barley, dates, and wheat bran) and green meal (Rhodes and dried alfalfa grasses), and the animals had free access to water and blocks of mineral salts. Over the course of the investigation, higher and lower temperatures were retroactively retrieved from https://weatherspark.com accessed on 17 May 2025. Using Google Maps, the locations of the farms included in the study were ascertained.

### 2.2. Ovarian Super-Stimulation Protocol

**Figure 1:**
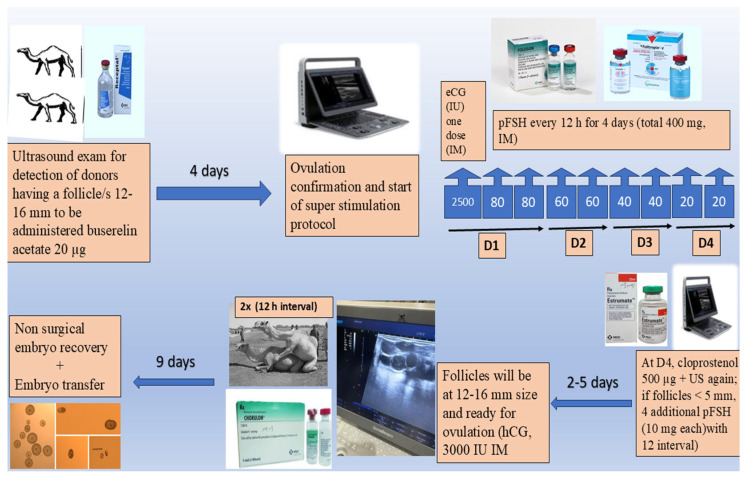
A diagram illustrating the steps of embryo transfer procedures in dromedary camels.

The superovulation protocol was first described by Skidmore et al. [3]. Transrectal B-mode ultrasonography (6–8 MHz linear transducer; SonoScape E1V, SonoScape Co., ShenZhen, China) was used to monitor ovarian dynamics. For donors’ ovarian super-stimulation, only females with at least one follicle (1.2–1.6 cm) were subjected to induced ovulation using 20 µg buserelin acetate (Receptal^®^, MSD Animal Health, Boxmeer, The Netherlands) and re-examined 4 days later to ascertain ovulation and follicle disappearance to start ovarian super-stimulation as follows (Figure 1). In detail, the first day began with an intramuscular single shot of equine chorionic gonadotropin (2500 IU; Folligon, MSD, Animal, Boxmeer, Health, The Netherlands) and double FSH (porcine type; 2 × 80 mg; Follitropin V, Bioniche Co., Belleville, ON, Canada) shots with a 12 h interval, followed by 2 × 60 mg, 2 × 40 mg, and 2 × 20 mg of pFSH on the 2nd, 3rd, and 4th days, respectively. Again, donors were examined to detect follicular dynamics, with a 5 mm follicular diameter as the landmark. In the case of follicles that did not reach this point, an additional 4 pFSH injections (10 mg each, 12 h intervals) were imposed. To let the follicles reach the ovulatory size, 500 μg cloprostenol (Estrumate^®^, MSD Animal Health, Boxmeer, The Netherlands) was injected on the 4th day from pFSH initiation. Normally, follicles need 2–5 days post-last pFSH dose to reach the ovulatory size (1.2–1.6 cm); once it was detected, donors were subjected to natural mating (twice, 12 h interval) with additional hCG administration (3000 IU; Chorulon^®^, MSD animal health, Boxmeer, The Netherlands) directly post-mating, aiding the ovulation process. Mating day was set to be the D0 to count on for embryo recovery day estimation.

### 2.3. Embryo Recovery

**Figure 2:**
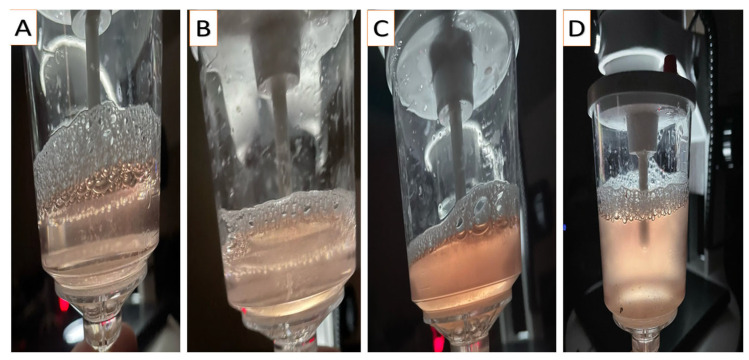
Camera photos showing the degree of turbidity in the flushing fluid, starting from no turbidity (clear; **A**) to low turbidity (grade 1; **B**), medium turbidity (grade 2; **C**), and high turbidity (grade 3; **D**).

**Figure 3:**
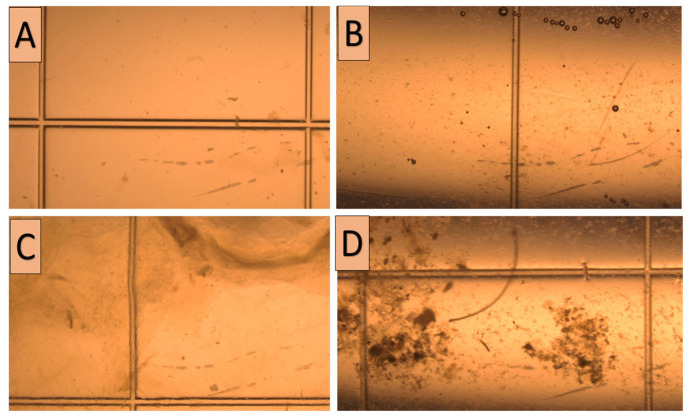
Stereo-microscopical photos (20×) showing the degree of debris in the flushing fluid during embryo searching, starting from no debris (clear; **A**) to low debris (grade 1; **B**), medium debris (grade 2; **C**), and high debris (grade 3; **D**).

**Figure 4:**
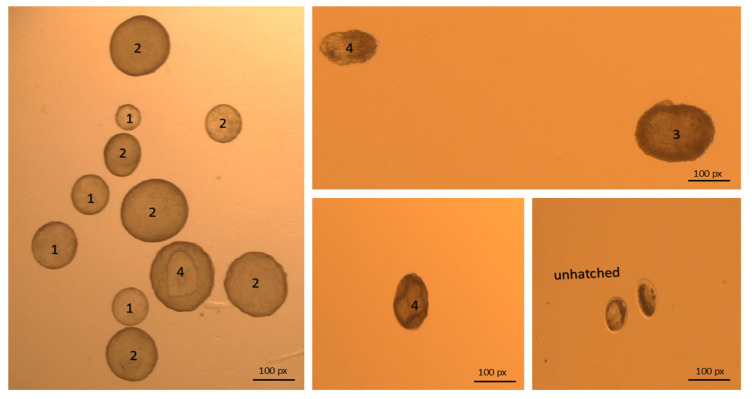
Stereo-microscopical photos (20×) illustrating the embryo quality grading as follows: Grade 1 (spherical + smooth surface + even trophectoderm), grade 2 (spherical + some irregularities in contour and cell color), grade 3 (dark patches + irregular contour + some protruded cells), and grade 4 (dark areas of degeneration and many extruded cells).

Following the procedures described by Anouassi and Tibary [4], embryos were recovered on D9 post-donor mating (to overcome delayed oviductal transport or asynchronous ovulation) using nonsurgical uterine flushing through the cervix while the donor was in a standing position. In a semi-dark area, the tail was wrapped and elevated, and the vulvo-perineal area was gauze-cleaned and thoroughly disinfected (70% ethanol). Through a Y-tubing catheter (18–22 gauge; Bioniche, Belleville, ON, Canada; introduced through the cervix, with its balloon inflated just after the internal os with 30–40 mL of air), both uterine horns were irrigated with jets (~50 mL each, with a sum of 0.5–1 L) of flushing media (Euroflush^®^, IMV Technologies, L’Aigle, France) and received by gravity into an embryo filter (IMV Technologies, L’Aigle, France) attached to a 1 L cylinder (to ascertain that all of the media was retrieved). Immediately after flushing ceased, the donor was administered 500 µg of cloprostenol (Estrumate^®^, MSD Animal Health, Boxmeer, The Netherlands) to ensure the lysis of any remaining corpora lutea. Collected media were graded for the turbidity (opacity) of the fluid subjectively using the score system (0–3), where 0 denotes clear fluid and 1, 2, and 3 denote low, medium, and high turbidity, respectively (Figure 2). Afterward, the fluid was examined for the existence of debris using a stereo microscope (20×; Wesco^®^, Western Scientific Co., Valencia, CA, USA). As for fluid turbidity, debris existence followed the same scoring system (Figure 3). The quality of embryos was classified according to Skidmore et al. [3]; the trophectoderm appearance, embryo shape, and transparency formed the judgment into grades 1, 2, 3, and 4 for excellent, good, poor, and folded, respectively (Figure 4). Only freshly collected hatched blastocysts (as morulae or early blastocysts with zona pellucida are considered arrested on D9) were transferred into synchronized recipients [4].

### 2.4. Embryo Transfer

For recipient–donor ovulation synchronization, we followed the protocol proposed by Anouassi and Tibary [4] that suits large-scale ET programs. Briefly, from a large recipient pool, those with a follicle(s) measuring 12–16 mm via rectal ultrasonography were administered 20 ug buserelin acetate, IM, a day after donor mating (−1 synchrony). Ovulation was confirmed on the ET day (D9 post donor mating) through rectal ultrasonography, and those with a corpus luteum (CL) more than 15 mm in size were used for ET. Recovered embryos were washed four times in a 5-well dish containing holding media (IMV Technologies, L’Aigle, France) and loaded (an embryo/recipient) into 0.25 mL French straws (media–air–media containing the embryo–air–media), followed by insertion of the ET gun with a straw. The recto-vaginal technique was adopted in this study. The ET gun protected with a sanitary sheath was inserted transvaginally through the cervix to the left uterine horn (2–4 cm cranial to the uterine bifurcation), where the embryo was placed [5,6,7,21]. Following ET, the recipients received extra attention, including proper diet (Rhodes (4 kg), wheat bran (3 kg), and alfalfa (0.5 kg)) and the opportunity to stand undisturbed, away from wind and noise to relieve the possible stressors on pregnancy establishment.

### 2.5. Assessment of EMs and Uterine Tone

**Figure 5:**
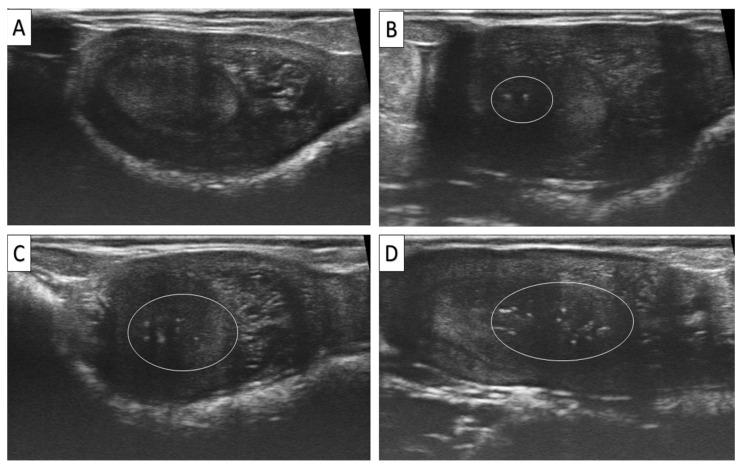
Dromedary recipients’ uterine ultrasonograms showing the existence of endometrial microcalcifications (EMs; hyperechoic foci, circled) on the embryo transfer day. These foci were classified into grades 0, 1, 2, and 3 for absence (**A**), low (1–2 foci; **B**), moderate (3–6 foci; **C**), and high (>6; **D**), respectively.

Rectal ultrasonography was adopted to detect the existence of hyperechoic foci (EMs, Figure 5). According to the number of foci, it was classified into grades 0, 1, 2, and 3 for absence, low (1–2 foci), moderate (3–6 foci), and high (>6), respectively [15]. Uterine tone was subjectively evaluated through rectal palpation of the uterine horns, and according to its rigidity, it was graded into 0, 1, 2, and 3 for soft, semi-rigid, rigid, and hard, respectively.

### 2.6. Pregnancy Diagnosis

Established pregnancy was diagnosed at two months after donor mating through rectal ultrasonography (6–8 MHz linear transducer; SonoScape E1V, SonoScape Co., China). The PR was calculated by dividing the number of camels that completed 2 months of pregnancy by the total number of transferred embryos × 100.

### 2.7. Statistical Analysis

Logistic regression analysis (binary) was used to set the significance level between the dependent variable (pregnancy at 2 months post-donor mating; not pregnant = 0 and pregnant = 1) and the independent variables (farm, location, embryo quality, flushing media turbidity and debris, and uterine morphometric factors (EMs and tone)) as categorical variables and high and low temperature as continuous numerical factors. The interaction between factors was assessed by adding the interaction equation in the syntax of the logistic regression analysis. Considering the large number of donors and bulls, the animal factor was not integrated as an independent factor. Version 25 of the SPSS statistical program was used to perform the analysis, with the significance point set at less than 0.05. The interpretation of the results was based on the exponential beta (Exp β; odds ratio) in the final fitted regression analysis model, where the value that exceeded or fell the 1 with *p* < 0.05 indicated an increase or decrease in the predictivity of the independent variables to affect the dependent variable, respectively. Spearman’s correlation coefficient was applied to draw the possible association between embryo quality and flushing fluid turbidity and debris. The number of recipients with a high number of EMs amounted to only 7 cases; therefore, they were not used for ET. In addition, the examined factors were not distributed randomly because of natural coincidence. However, location was controlled, and all embryos in the different classes could have been randomly assigned and transferred to two locations, even across five farms. This lack of randomization may affect the results of the present study; however, the large number of transferred embryos and the fact that most of the procedures (78%) were performed in one location could dilute the negative effect.

## 3. Result

**Figure 6:**
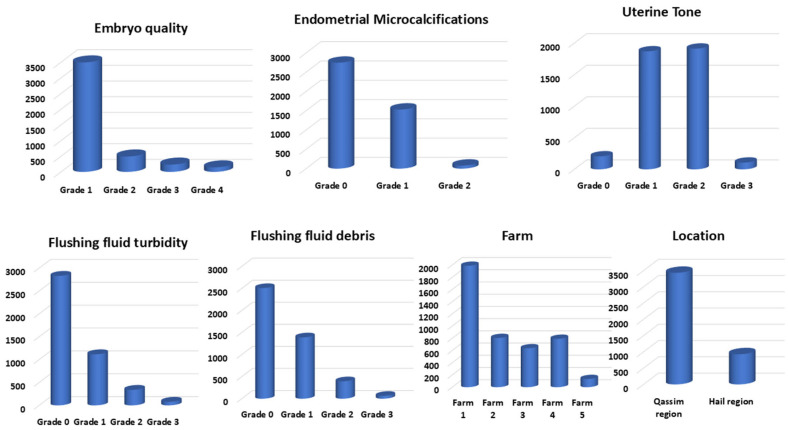
Frequencies of different degrees of the examined factors (n = 4360) in the camel embryo transfer commercial program (season 2022–2023).

**Figure 7:**
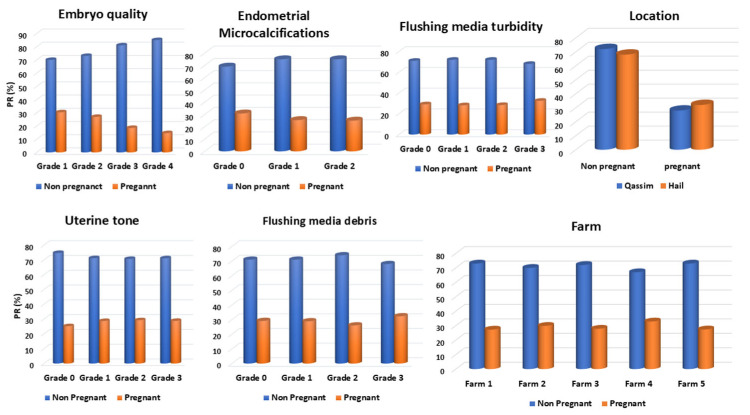
Distribution of all examined factors in relation to different farms in the dromedary camel embryo transfer program (n = 4360) season 2022–2023.

**Figure 8:**
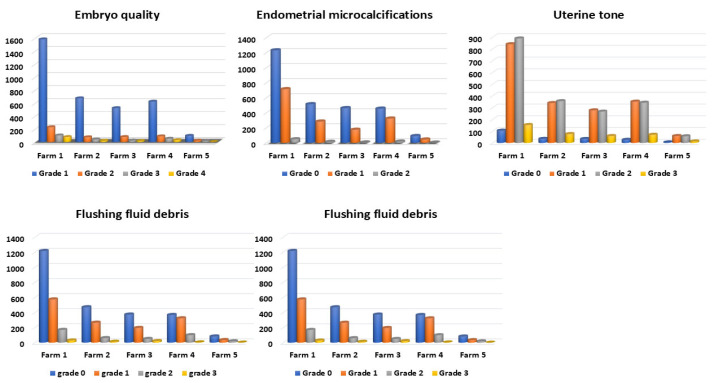
Pregnancy rate in relation to different degrees of the examined factors (n = 4360) in the camel embryo transfer commercial program (season 2022–2023).

**Figure 9:**
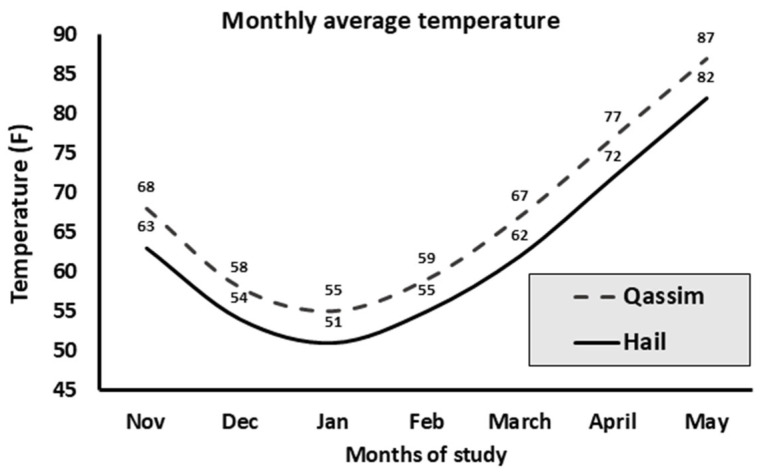
Monthly average environmental temperature (high and low) in Qassim and Hail regions during the experimental months of embryo transfer (2022–2023; n = 4360). Data were retrieved from https://weatherspark.com accessed on 17 May 2025.

The 2-month pregnancy rate obtained was 28.83% (1257/4360). The examined factors and their categories’ distribution are presented in Figure 6. Factors/farm distribution is presented in Figure 7. PR/factors and their categories are summarized in Figure 8. Temperature changes throughout the season (2022–2023) in the Qassim and Hail regions are presented in Figure 9.

### 3.1. Effect of Uterine Factors (EMs and Tone) on PR

The two-month pregnancy rate in dromedary ET (Table 1) was not affected by recipients’ uterine tone (*p* = 0.648), but it was affected by EMs (*p* < 0.01). In detail, the PR odds decreased merely by 1.3 times when the embryos were transferred to a low- (odds ratio = 0.768; confidence intervals: 0.667–0.885, *p* < 0.01) or medium EM (odds ratio = 0.751; confidence intervals: 0.449–1.255, *p* < 0.05) uterine parenchyma compared to an EM-free uterus. There was no significant difference between different degrees of uterine EMs (odds ratio = 1.092; confidence intervals: 0.655–1.819) on the PR.

### 3.2. Effect of Embryo Quality on PR

Embryo grading predicted the pregnancy rate in ET in dromedaries at 2 months post-donor mating (*p* < 0.01; Table 1). Specifically, compared to grade 1 embryos, transferring grade 3 (odds ratio = 0.535; confidence intervals: 0.381–0.752, *p* < 0.01) and 4 (odds ratio = 0.378; confidence intervals: 0.236–0.604, *p* < 0.01) embryos reduced the 2-month pregnancy odds by 1.9 and 2.6 times, respectively. There was no significant difference between transferring either grade 1 or 2 embryos on pregnancy odds (odds ratio = 0.846; confidence intervals: 0.684–1.046).

### 3.3. Effect of Flushing Fluid Debris and Turbidity on Embryo Quality and PR

Following embryo recovery in the dromedary ET program, neither the flushing media turbidity nor debris existence had a significant effect on 2-month pregnancy completion post-donor mating (Table 1). There was a very weak significant correlation (Table 2) between both flushing fluid turbidity (r = 0.033; *p* < 0.01) and debris (r = 0.058; *p* < 0.01) and embryo quality.

**Table 1:** Number of embryos transferred (n = 4360) separated by factors and categories and the corresponding 2-month pregnancy rate (season 2022–2023) using a binary logistic regression analysis model.

	Examined Factor	Classification Categories	Frequencies (%)	Pregnancy Rate (%)	Confidence Interval (95%)	Odds Ratio	*p*-Value
1	Uterine tone	Total					NS
		Grade 0	206 (4.7)	52/206 (25.24)		Indicator	
		Grade 1	1870 (42.9)	538/1870 (28.77)	0.840–1.645	1.176	NS
		Grade 2	1913 (43.9)	560/1913 (29.27)	0.843–1.656	1.182	NS
		Grade 3	107 (8.5)	107/371 (28.84)	0.709–1.576	1.057	NS
2	Endometrial Microcalcifications	Total					<0.01
		Grade 0	2747 (63)	847/2747 (30.83)		Indicator	
		Grade 1	1533 (35.2)	390/1533 (25.44)	0.667–0.885	0.768	<0.01
		Grade 2	80 (1.8)	20/80 (25.00)	0.449–1.255	0.751	<0.05
3	Flushing fluid turbidity	Total					NS
		Grade 0	2829 (64.9)	822/2829 (29.05)		Indicator	
		Grade 1	1116 (25.6)	314/1116 (28.13)	0.834–1.137	0.974	NS
		Grade 2	338 (7.8)	96/338 (28.40)	0.782–1.314	1.014	NS
		Grade 3	77 (1.8)	25/77 (32.46)	0.697–1.966	1.170	NS
4	Flushing fluid debris	Total					NS
		Grade 0	2509 (57.5)	731/2509 (29.13)		Indicator	
		Grade 1	1391 (31.9)	402/1391 (28.90)	0.874–1.172	1.012	NS
		Grade 2	395 (9.1)	103/395 (26.07)	0.673–1.113	0.865	NS
		Grade 3	65 (1.5)	21/65 (32.30)	0.627–1.921	1.097	NS
5	Embryo quality	Total					<0.01
		Grade 1	3493 (80.1)	1061/3493 (30.37)		Indicator	
		Grade 2	491 (11.3)	132/491(26.88)	0.684–1.046	0.846	NS
		Grade 3	232 (5.3)	43/232 (18.53)	0.381–0.752	0.535	<0.01
		Grade 4	144 (3.3)	21/144 (14.58)	0.236–0.604	0.378	<0.01
6	Farm	Total					<0.05
		Farm 1	1989 (45.6)	543/1989 (27.30)		Indicator	
		Farm 2	807 (18.5)	241/807 (29.86)	0.947–1.358	1.134	NS
		Farm 3	638 (14.6)	178/638 (27.89)	0.844–1.258	1.030	NS
		Farm 4	793 (18.2)	261/793 (32.91)	1.095–1.564	1.309	<0.05
		Farm 5	135 (3.1)	37/135 (27.40)	0.659–1.451	0.978	NS
7	Location	Qassim region	3432 (78.7)	959/3432 (27.94)		Indicator	
		Hail region	928 (21.3)	298/928 (32.11)	1.056–1.469	1.246	<0.01
8	Temperature	High			0.978–1.018	0.998	NS
		Low			0.985–1.036	1.010	NS

NS = non-significant.

**Table 2:** Correlation between embryo quality and flushing fluid turbidity and debris (n = 4360) in the dromedary embryo transfer program (season 2022–2023).

Embryo Quality	Debris	Turbidity			
Embryo quality	Correlation Coefficient		1.000	0.058 **	0.033 *
	Significance		.	<0.01	<0.05
Debris	Correlation Coefficient		0.058 **	1.000	0.248 **
	Significance		0.000	.	<0.01
Turbidity	Correlation Coefficient		0.033 *	0.248 **	1.000
	Significance		0.031	<0.01	.

* *p* < 0.05; ** *p* < 0.01.

### 3.4. Location Effect on PR

The region where the dromedary ET program was applied had a significant effect on PR attainment (*p* < 0.01; Table 1). In Saudi Arabia, compared to the Qassim region, the establishment of an ET program in the Hail region increased the likelihood of recipients attaining the PR by ~1.2 times ((odds ratio = 1.309; confidence intervals: (odds ratio = 1.246; confidence intervals: 1.095–1.564, *p* < 0.01).

### 3.5. Farm Effect on PR

The was an effect of farm on the PR likelihood in dromedary ET (*p* < 0.05; Table 1). Compared to farm 1, higher pregnancy odds were recorded in farm 4 (odds ratio = 1.309; confidence intervals: 1.095–1.564, *p* < 0.05). There was no significant difference between farm 1 pregnancy odds and those of farm 2 (odds ratio = 1.134; confidence intervals: 0.947–1.358), 3 (odds ratio = 1.030; confidence intervals: 0.844–1.258), and 5 (odds ratio = 0.987; confidence intervals: 0.659–1.451).

### 3.6. Temperature Effect on PR

There was no significant effect of temperature, either high or low, on PR (Table 1).

### 3.7. Effect of Interaction Between All Examined Factors on PR

Only interaction between high temperature and embryo quality had a significant effect (*p* < 0.05) on PR. Specifically, high temperature decreased the pregnancy rate only when grade 4 embryos were transferred (odds ratio = 0.951; confidence intervals: 0.902–1.002, *p* < 0.05). Conversely, other embryo grades were not affected by temperature changes.

## 4. Discussion

The total PR, in this study, appears to be lower than other published works [5,6,7], although it is still within the previously reported ET success rate for camelids (21–42%; [4]). This difference could be attributed to the number of transferred embryos (4360 vs. 2095 [6], 1309 [5], and 377 [7]). Furthermore, in contrast with other studies that used only normal uterine conditions [6], only high-quality embryos [7], and only one location [5,6,7], the current study looked at factors related to recipients’ uterine quality (EMs), different embryo quality, and locations (different environmental conditions) that might result in a decrease in the PR. In addition, factors related to the donor, bull, and recipients (age and body condition score) might contribute to ET outcomes [4,8,12].

In this study, transferring embryos to recipients with a low or moderate degree of EMs resulted in a 1.3 times lower likelihood of PR than those without EMs. This is the first study to elaborate on the association between EMs and PR in dromedary camel ET programs, which could refine the recipient’s selection protocols and enhance the ET success rate. Indeed, EMs are associated with recurrent pregnancy loss [13] and infertility [22] in humans. Despite the difficulties in explaining the influence of EMs on PR due to the lack of available information in camels, several factors have been noted to be associated with the existence of EMs in humans, such as endometrial aging [15], chronic endometrial degenerative diseases [14], and endometrial trauma or chronic endometritis [13]. The general view is that EMs lead to a decrease in endometrial activity needed for embryo development, nutrition, and implantation [23], which is in line with our findings after ET in dromedary camels.

The present results revealed that there was no effect of uterine tone on the PR. Fernandes [20] concluded that a semi-flaccid uterus is better than a rigid uterus for embryo implantation in bovine ET. The low number of recipients used (29 recipients) and the species difference (bovines vs. camelids) might have affected the conclusions of the other study. Uterine tonus is largely controlled by the E_2_/P_4_ ratio. P_4_ inhibits E_2_ receptors in myometrial and epithelial cells. When there is sound CL development, a low muscle tonus is observed after rectal palpation; alternatively, when abnormal/delayed CL development occurs, the P_4_ concentration will be low, and the uterine tonus will be higher, depending on follicular development. In bovines, CL volume and luteal P_4_ production during the mid-luteal phase have a strong correlation but not with circulating P_4_ concentrations [24,25]. For instance, this link was found to be non-significant on Day 8 of the estrous cycle [26], underscoring the significance of the interaction between variables other than CL size, such as hepatic clearance and diet, on P_4_ plasma concentrations [27,28].

The regression analysis model also identified embryo quality as a predictive factor for the PR after ET in dromedaries. Recipients who received grade 3 or 4 embryos had a lower likelihood of attaining the PR, by 1.9 and 2.6 times, respectively. The PR following ET is impacted by the embryo grading used in this study, which also aids in future improvements to camel ET methods for improved results. Many studies in camels [4,6,7] and alpacas [8] assured a high PR following ET with high-grade vs. low-grade embryos. Embryo shape, transparency, and tropho-ectodermal appearance are considered as the main determinants of embryo quality in camelids [3]. It has been stated that low PRs after ET and high pregnancy loss were recorded with the transfer of dark, elongated, and spindle embryos in dromedaries [7]. Embryo darkness denotes the existence of dead blastomeres and viability loss [6]. High morphological grade embryos have a greater ability to initiate maternal recognition crosstalk, endometrial implantation, and developmental competence [29]. Since embryos must secrete estradiol 17β and estrone sulphate for maternal recognition and luteolysis inhibition [29], low-grade embryos may be unable to secrete a sufficient amount of estrogens to prevent luteolysis. Conversely, in a small number of transferred embryos in dromedaries (n = 139), Ararooti et al. [30] claimed that transferring either round, oval, or collapsed embryos did not affect the 33-day pregnancy rate. Compared to the embryos (n = 4360) transferred in our study, it seems that the low number of transferred embryos in the other study might be the reason for the difference. Regarding hatched blastocyst transfer, Anouassi and Tibary [4] concluded that early blastocysts with a zona pellucida or normal-looking morulae that are recovered from the uterus are deemed to be arrested and non-transferable. Although, McKinnon et al. [31] recorded a 26% PR with morulae and early blastocysts. However, in our protocol of D9 embryo flushing, morulae and unhatched blastocysts may be arrested, as this developmental stage fits with D6.5-D7 after mating [31].

We found no effect of flushing fluid turbidity or debris on the likelihood of establishing PR in dromedary ET. This alteration in flushing fluid transparency might be due to normal detachment of the endometrial lining containing the implanted embryos [11]. We also found no effect of flushing fluid turbidity or debris on the quality or developmental competence of the recovered embryos compared to the clear flushing fluid; this may explain the non-significant effect on PR with medium or high turbidity or debris-containing flushing fluid.

The location and the farm where this ET program was implemented had a significant impact on the PR. In Saudi Arabia, compared to the Qassim region, the application of an ET program in the Hail region (farms 4 and 5) increased the likelihood of recipients completing 2-month pregnancies by ~1.2 times. PR difference between farms could be related to many factors such as technicians, animals (donor and recipients) criteria, nutrition, and environmental conditions [4,32]. There is an important factor to be considered, namely, “the stress”. Qassim’s main farms were related to the commercial ET center, where the donors and recipients were transferred from their homes to the ET center through vehicles or walking for hours, in addition to a long period of acclimation and fear. In the Hail region, the ET procedures were performed on the owner’s farms using recipients that already belonged to the same farm (no additional stress). It has been concluded that stress, especially prolonged, diminishes reproductive performance, conception rates after ET, and artificial insemination [33,34]. Since stress biomarkers were not tested in this study (a limiting factor), it is advised that stress be assayed when farms and location are considered as contributing factors affecting the PR after ET in dromedaries.

Heat stress induces low sperm motility and higher abnormalities, impairs oocyte development and fertilization, and delays embryonic development [35]. In this study, we found no effect of temperature changes on the PR throughout the season. However, there was an interaction between higher temperature and PR with low-quality embryos. Similar outcomes have been obtained in bovines [36,37]. Indeed, heat tolerance differs between sperm, oocytes, early embryos, and peri-implantation blastocytes, with the latter being able to tolerate higher ranges of temperature, especially with higher quality embryos, considering the role of trophectoderm in anti-thermal protection [29,30]. In addition, it has been reported that conception rate following ET is affected by summer heat stress but not by artificial insemination in bovines [38,39]. Camels are more suited to hot, dry climates than cattle because of their superior thermal homeostasis. They can withstand significant daily temperature changes, helping them conserve water. Camels’ thick fur protects them from the heat, and they sweat more effectively and only when needed. Additionally, they have a unique nasal system that reduces water loss when they breathe. Cattle, on the other hand, sweat more easily, maintain a narrow temperature range, and lose more water through evaporation and respiration. Compared to cattle, camels are far more adept at handling heat stress and water scarcity because of these adaptations [40,41].

## 5. Conclusions

In the dromedary camel ET program, farm location, EM, and embryo quality all had an impact on the chance of PR establishment. Transferring grade 1 or 2 embryos in an EM-free uterus in the Hail region (Saudi Arabia) increased the chance of PR. Additional research on embryo categorization and EM should be conducted.

## Data Availability

Data availability is possible upon request.

## References

[1] Skidmore J.A. (2011). Reproductive physiology in female old world camelids. Anim. Reprod. Sci..

[2] Tibary A., Anouassi A., Actes (1997). Theriogenology in Camelidae: Anatomy, Physiology, Pathology and Artificial Breeding.

[3] Skidmore J.A., Billah M., Allen W. (2002). Investigation of factors affecting pregnancy rate after embryo transfer in the dromedary camel. Reprod. Fertil. Dev..

[4] Anouassi A., Tibary A. (2013). Development of a large commercial camel embryo transfer program: 20 years of scientific research. Anim. Reprod. Sci..

[5] Karen A., Mansour N. (2020). Factors affecting pregnancy rates and pregnancy losses after embryo transfer in dromedary camels. Anim. Reprod. Sci..

[6] Karen A., Abd-Elfattah A., Nasef M., Rahman R.U., Ihsan M.B., Muthukumaran S. (2021). Factors affecting outcomes of embryo transfer in dromedary camels: A retrospective study. Reprod. Domest. Anim..

[7] Abd-Elfattah A., Agag M., Nasef M., Muthukumaran S., El-Raey M., El-Khawaga A., Karen A. (2020). Preservation of dromedary camel embryos at 4° C for up to 5 days: Factors affecting the pregnancy and pregnancy loss rates. Theriogenology.

[8] Vaughan J., Mihm M., Wittek T. (2013). Factors influencing embryo transfer success in alpacas—A retrospective study. Anim. Reprod. Sci..

[9] Pérez-Marín C.C., Vizuete G., Borge C., Galisteo J.J. (2018). Cytological and bacteriological sampling from filters used for embryo recovery to evaluate the uterine status of donor mares. Acta Vet. Hung..

[10] Mulligan B.P., Skidmore J.A. (2023). A comparison of culture and cooling for the short term preservation of in vivo derived dromedary camel embryos of varying morphological quality. Theriogenology.

[11] Gilbert O.R. (2012). The effects of endometritis on the establishment of pregnancy in cattle. Reprod. Fertil. Dev..

[12] Sumar J.B. (2013). Embryo transfer in domestic South American camelids. Anim. Reprod. Sci..

[13] Feyles V., Moyana T.N., Pierson R.A. (2000). Recurrent pregnancy loss associated with endometrial hyperechoic areas (endometrial calcifications): A case report and review of the literature. Clin. Exp. Obstet. Gyn..

[14] Walter I., Helmreich M., Handler J., Aurich C. (2003). Mineralised deposits in the uterine glands of mares with chronic endometrial degeneration. Vet. Rec..

[15] Truskinovsky A.M., Gerscovich E.O., Duffield C.R., Vogt P.J. (2007). Endometrial Microcalcifications Detected by Ultrasonography: Clinical Associations, Histopathology, and Potential Etiology. Int. J. Gyn. Pathol..

[16] Bonafos L.D., Carnevale E.M., Smith C.A., Ginther O.J. (1994). Development of uterine tone in nonbred and pregnant mares. Theriogenology.

[17] Porter D.G., Berham H.R. (1971). Prostaglandin-induced myometrial activity inhibited by progesterone. Nature.

[18] Rodriguez-Martinez H., Mckenna D., Weston P.G., Whitmore H.L., Gustafsson B.K. (1987). Uterine motility in the cow during the estrous cycle. I. Spontaneous activity. Theriogenology.

[19] Guimarães C.R., Seber M.F., Neto J.D., Fernandes C.A., Rodrigues É.P., Torres B.A., Moreira Viana J.H., Palhão M.P. (2024). Uterine tone: A ne-447 glected criterion for the selection of bovine embryo transfer recipients. Reprod. Domest. Anim..

[20] Fernandes C.A. (1999). Inovulações não cirúrgicas e taxa de gestação de receptoras de embrião. Arq. Bras. Med. Veterinária Zootec..

[21] Tinson A., Sambyal R., McCallum C. Factors affecting embryo recovery in dromedary camels: Review of results over the last 20 yrs. Proceedings of the ICAR Satellite Meeting on Camelid Reproduction.

[22] Pereira M.C., Vaz M.M., Miranda S.P., Araújo S.R., Menezes D.B., das Chagas Medeiros F. (2014). Uterine cavity calcifications: A report of 7 cases and a systematic literature review. J. Minim. Invasive Gyn..

[23] AbdullGaffar B., AlMulla A. (2020). Endometrial calcifications. Int. J. Surg. Pathol..

[24] Herzog K., Brockhan-Lüdemann M., Kaske M., Beindorff N., Paul V., Niemann H., Bollwein H. (2010). Luteal blood flow is a more appropriate indicator for luteal function during the bovine estrous cycle than luteal size. Theriogenology.

[25] Lüttgenau J., Bollwein H. (2014). Evaluation of bovine luteal blood flow by using color Doppler ultrasonography. Reprod. Biol..

[26] Mann G.E., Lamming G.E. (2009). Relationship between maternal endocrine environment, early embryo development and inhibition of the luteolytic mechanism in cows. Reproduction.

[27] Butler W.R. (2000). Nutritional interactions with reproductive performance in dairy cattle. Anim. Reprod. Sci..

[28] Rabiee A.R., Dalley D., Borman J.M., Macmillan K.L., Schwarzenberger F. (2002). Progesterone clearance rate in lactating dairy cows with two levels of dry matter and metabolisable energy intakes. Anim. Reprod. Sci..

[29] Skidmore J., Allen W., Heap R. (1994). Oestrogen synthesis by the peri-implantation conceptus of the one-humped camel 459 (Camelus dromedarius). J. Reprod. Fertil. Suppl..

[30] Ararooti T., Niasari-Naslaji A., Asadi-Moghaddam B., Razavi K., Panahi F. (2018). Superovulatory response following FSH, eCG-FSH and hMG and pregnancy rates following transfer of hatched blastocyst embryos with different diameter and shape in dromedary camel. Theriogenology.

[31] McKinnon A.O., Tinson A.H., Nation G. (1994). Embryo transfer in dromedary camels. Theriogenology.

[32] Ferraz P.A., Burnley C., Karanja J., Viera-Neto A., Santos J.E.P., Chebel R.C., Galvão K.N. (2016). Factors affecting the success of a large embryo transfer program in Holstein cattle in a commercial herd in the southeast region of the United States. Theriogenology.

[33] Dobson H., Tebble J.E., Smith R.F., Ward W.R. (2001). Is stress really all that important?. Theriogenology.

[34] Cooke R.F., Bohnert D.W., Cappellozza B.I., Mueller C.J., Delcurto T. (2012). Effects of temperament and acclimation to handling on reproductive performance of Bos taurus beef females. J. Anim. Sci..

[35] Hansen P.J. (2009). Effects of heat stress on mammalian reproduction. Philos. Trans. R. Soc. B Biol. Sci..

[36] Lee J., Lee S., Ryu G., Kim D., Baek H.U., Kim J., Lee K., Kim S., Kim S., Dang C.-G. (2024). A retrospective analysis of conception per embryo transfer in dairy cattle in South Korea. Theriogenology.

[37] Chebel R.C., Dem’etrio D.G.B., Metzger J. (2008). Factors affecting success of embryo collection and transfer in large dairy herds. Theriogenology.

[38] Putney D.J., Drost M., Thatcher W.W. (1988). Embryonic development in superovulated dairy cattle exposed to elevated ambient temperatures between Days 1 to 7 post insemination. Theriogenology.

[39] Ambrose J.D., Drost M., Monson R.L., Rutledge J.J., Leibfried-Rutledge M.L., Thatcher M.J., Kassa T., Binelli M., Hansen P.J., Chenoweth P.J. (1999). Efficacy of timed embryo transfer with fresh and frozen in vitro produced embryos to increase pregnancy rates in, heat-stressed dairy cattle. J. Dairy Sci..

[40] Lees A.M., Sejian V., Wallage A.L., Steel C.C., Mader T.L., Lees J.C., Gaughan J.B. (2019). The impact of heat load on cattle. Animals.

[41] Fesseha H., Desta W. (2020). Dromedary camel and its adaptation mechanisms to desert environment: A review. Int J. Zool. Stu..

